# Supplementary material for: The Systemic Redox Status Is Maintained in Non-Smoking Type 2 Diabetic Subjects Without Cardiovascular Disease: Association with Elevated Triglycerides and Large VLDL
Source: J Clin Med. 2019 Dec 24;9(1):49. doi: 10.3390/jcm9010049 (PMC7019670; doi:10.3390/jcm9010049)
Supplement: Supplementary file 1 [file jcm-09-00049-s001.pdf]

Supplemental material

Table 1. Univariate relationships of plasma free thiols with clinical and laboratory variables in males (n = 98) and females (n = 70).

	Males	Females
Age	-0.333 *	-0.049
Systolic blood pressure	-0.069	-0.001
Diastolic blood pressure	0.055	-0.011
BMI	0.091	0.035
Waist	0.069	0.046
Glucose	0.185	0.197
HbA1c	0.103	0.193
Insulin	0.196	-0.006
HOMA-IR	0.222 *	0.058
eGFR	0.270 **	0.074
Total cholesterol	0.103	0.122
Non-HDL cholesterol	0.160	0.166
LDL cholesterol	-0.009	0.103
HDL cholesterol	-0.206 *	-0.112
Triglycerides	0.351***	0.149
ApoB	0.106	0.135
VLDL-P	0.299 *	0.049
Large VLDL	0.294 **	0.199
Medium VLDL	0.270 **	0.144
Small VLDL	0.119	-0.168
FFA	-0.132	-0.100
PLTP activity	0.107	0.024
Adiponectin	-0.245 ***	-0.138

Pearson correlation coefficients are shown. Non-parametrically distributed data are $\log_e$ transformed. **P*<0.05; ***P*≤0.01: ****P*≤0.001. Abbreviations: ApoB: apolipoprotein B; BMI: body mass index; e-GFR: estimated glomerular filtration rate; FFA: free fatty acids: HbA1c: glycated hemoglobin; HDL: high density lipoproteins; LDL: low density lipoproteins; PLTP: phospholipid transfer protein; VLDL: very low density lipoproteins: VLDL-P: very low density particle concentration.

Table 2. Multivariable linear regression analysis demonstrating independent associations of plasma free thiols with age and individual metabolic syndrome components in males (n=98) and females (n=70).

	Males		Females	
	**β**	***P*-value**	**β**	***P*-value**
Age	- 0.363	0.001	-0.027	0.843
Elevated glucose	0.072	0.492	0.088	0.498
Elevated blood pressure	0.029	0.782	-0.193	0.178
Enlarged waist	0.009	0.932	-0.004	0.978
Elevated triglycerides	0.261	0.018	0.186	0.177
Low HDL cholesterol	-0.071	0.519	0.183	0.172

Abbreviations: β: standardized regression coefficient; HDL: high density lipoproteins.

Table 3. Multivariable linear regression analysis demonstrating independent associations of plasma triglycerides with age, sex, free thiols, free fatty acids, PLTP activity and adiponectin and glucose in males (n = 98) and females (n = 70).

	Males	Females

	β	*P*-value	β	*P*-value
Age	-0.029	0.753	0.045	0.723
Free thiols	0.251	0.007	0.136	0.254
FFA	0.188	0.032	0.131	0.293
PLTP activity	0.230	0.009	0.266	0.044
Adiponectin	-0.332	0.000	-0.223	0.085
Glucose	0.109	0.234	-0.038	0.800

Triglycerides and adiponectin are $\log_e$ transformed. Abbreviations: β: standardized regression coefficient; FFA: free fatty acids: PLTP phospholipid transfer protein.

Table 4. Multivariable linear regression analysis demonstrating independent associations of large very low density lipoprotein subfractions with age, sex, free thiols, free fatty acids, PLTP activity, adiponectin and glucose in males (n=98) and females (n=70).

	**Males**		**Females**	
	β	*P*-value	β	*P*-value
Age	-0.018	0.882	-0.023	0.872
Free thiols	0.184	0.136	0.188	0.140
Free fatty acids	0.206	0.079	0.302	0.031
PLTP	0.206	0.072	0.100	0.493
Adiponectin	-0.346	0.003	-0.204	0.140
Glucose	0.147	0.226	0.023	0.889

VLDL subfractions and adiponectin are $\log_e$ transformed. Abbreviations: β: standardized regression coefficient; FFA: free fatty acids: PLTP phospholipid transfer protein; VLDL: very low density lipoproteins.
